# T cell stimulation and expansion by SunTag-based clustering of anti-CD3/CD28 scFv

**DOI:** 10.18632/aging.103318

**Published:** 2020-06-10

**Authors:** Kunhong Zhong, Zhiyong Liu, Hongjian Li, Shasha Zhao, Yuelong Wang, Wenhao Guo, Xi Zheng, Hui Yang, Gang Guo, Liangxue Zhou, Jianguo Xu, Aiping Tong

**Affiliations:** 1 State Key Laboratory of Biotherapy and Cancer Center, West China Hospital, West China Medical School, Sichuan University, Chengdu, Sichuan Province, China; 2 Department of Neurosurgery, West China Hospital, Sichuan University, Chengdu, China; 3 Lung Cancer Center, West China Hospital, Sichuan University, Chengdu, China; 4 Department of Otolaryngology, Head and Neck Surgery, West China Hospital, Sichuan University, Chengdu, China

**Keywords:** T cell stimulation, T cell expansion, SunTag, CAR-T

## Abstract

Therapeutic *ex vivo* T cell expansion is limited by low rates and poor functionality, especially for T cells from aged cancer patients. Here, we describe a novel method for T cell stimulation and expansion using a system named SunTag-based clustering of anti-CD3/CD28 scFv (SBCS). In this method, SunTag was used to recruit up to 13 copies of anti-CD3/CD28 scFv for T cell activation. Compared with the traditional method using immobilized CD3/CD28 antibodies, the SBCS system produced approximately 1.5-fold greater expansion of T cells from healthy donors, and more than 2-fold greater expansion of T cells from aged cancer patients after stimulation. The efficiency of expansion depended mainly on the concentration of the clustered polymers of anti-CD3 scFv rather than anti-CD28 scFv. We also demonstrated that the SBCS-expanded T cells could be used to prepare functional chimeric antigen receptor modified T cells for antitumor therapy.

## INTRODUCTION

Immunotherapy mediated by T cells has a high potential to treat various diseases [1]. Moreover, T cell-based therapies have shown unprecedented success in the treatment of cancers. For instance, chimeric antigen receptor modified T cell (CAR-T) therapy against CD19 is effective in treating B-cell acute lymphoblastic leukemia [2, 3]. The rapid expansion of functional T cells *in vitro* (a primary step for such therapies) remains a challenge, especially for T cells from aged cancer patients. Previous research has shown that T cell activation requires three signals, namely T cell receptor (TCR) stimulation, TCR costimulation, and prosurvival cytokine signaling [4]. T cell stimulus intensity depends on the density of bound receptors in contact with T cells [5], and higher receptor density contributes to better T cell activation [6].

Currently, the CD3/CD28 antibodies and microbeads (Dynabeads) functionalized with activating antibodies for CD3 and CD28 are used to activate and expand T cells *in vitro* [7, 8]. Although they contribute to T cell expansion, there are certain limitations. CD3/CD28 antibodies are immobilized to plastic surfaces for better function [9]; however, low rates of expansion remain. As one of the most commonly used systems for T cell expansion, Dynabeads are non-degradable and must be separated from the cell product prior to infusion, which can increase cost [10]. Furthermore, Dynabeads are prone to sink to the bottom of culture dishes. Therefore, the rate of T cell expansion stimulated by Dynabeads is low under stationary culture conditions.

SunTag, a tandem repeat of multiple copies of the 19 amino-acid GCN4 peptide separated by amino acid linkers of 5 amino acid residues, is able to recruit effector domains fused to a single-chain variable fragment (scFv) against GCN4 (αGCN4scFv). Thus far, the SunTag system has mainly been used for intracellular imaging or DNA editing via its signal amplification ability [11–15].

In the present study, we hypothesized that anti-CD3scFv polymers and anti-CD28scFv polymers clustered by SunTag can be used for T cell expansion. Thus, we developed SunTag-based clustering of anti-CD3/CD28 scFv (SBCS) for stimulating T cells *in vitro*. Moreover, we used SBCS-expanded cells to prepare the B7-H3-specific chimeric antigen receptor T cells (B7-H3 CAR-T cells), and evaluated the tumor-killing effect of B7-H3 CAR-T cells against head and neck cancer cell (HNC) line FaDu and cervical cancer cell line Hela. Our results demonstrated that the SBCS system can efficiently expand T cells, especially T cells from aged cancer patients.

## RESULTS

### Expression and purification of recombinant proteins

**Figure 1:**
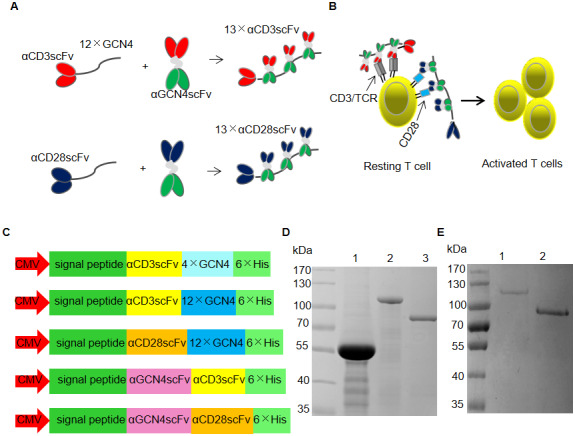
**SunTag-based clustering of αCD3/CD28 scFv (SBCS).** (**A**, **B**) Schematic of the SBCS strategy for stimulation and expansion of T cells. (**A**) αCD3scFv or αCD28scFv fused with 12 tandem copies of the GCN4 tag was used to recruit αCD3/CD28 scFv fused with αGCN4scFv, forming 13×αCD3scFv or 13×αCD28scFv, respectively. (**B**) 13×αCD3scFv and 13×αCD28scFv bind to the TCR/CD3 complex and the CD28 molecule, respectively, to activate resting T cells. (**C**) Schematic drawing of the vectors used for recombinant protein expression. (**D**, **E**) SDS-PAGE analysis of the purified recombinant proteins of (**C**). All recombinant proteins were expressed by transient transfection of HEK293FT cells. Lanes 1, 2, and 3 of (D) represent αCD3scFv-4×GCN4, αCD3scFv-12×GCN4, and αGCN4scFv-αCD3scFv, respectively. Lanes 1 and 2 of (**E**) represent αCD28scFv-12×GCN4 and αGCN4scFv-αCD28scFv, respectively.

Figure 1A and 1B show the schemes of the formation of αCD3/CD28 scFv polymers for T cell expansion. αCD3scFv or αCD28scFv was recruited by 12 tandem copies of GCN4 to form 13×αCD3scFv or 13×αCD28scFv. Recombinant proteins were expressed by transient transfection into HEK293FT cells with vectors containing CMV promoters (Figure 1C). The purified proteins were analyzed using sodium dodecyl sulfate polyacrylamide gel electrophoresis (SDS-PAGE; Figure 1D and 1E). Lanes 1, 2, and 3 in Figure 1D represent αCD3scFv-4×GCN4, αCD3scFv-12×GCN4, and αGCN4scFv-αCD3scFv, respectively. Lanes 1 and 2 of Figure 1E represent αCD28scFv-12×GCN4 and αGCN4scFv-αCD28scFv, respectively. We also prepared 18× and 24×αCD3scFv for T cell expansion. Although their activities were greater, the expression level was too low to prepare (data not shown).

### Proliferation of primary human T cells isolated from healthy donors

**Figure 2:**
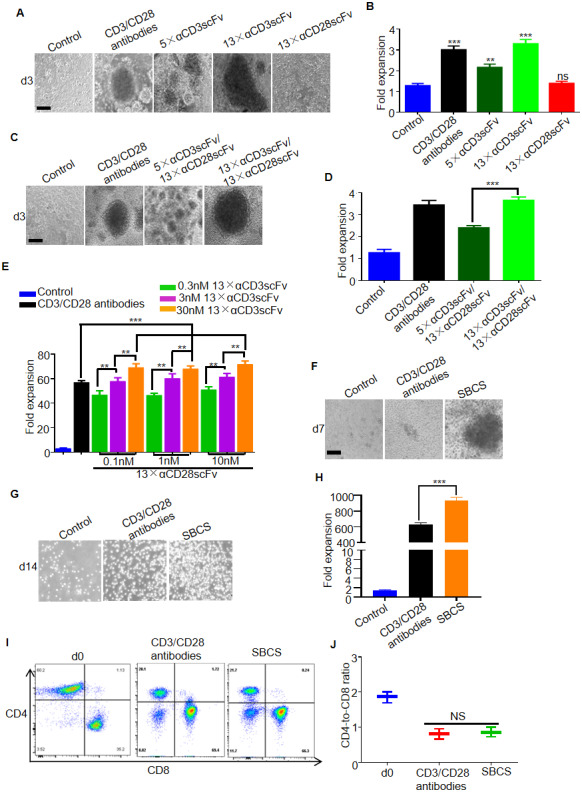
**Expansion of T cells from healthy donors.** (**A**) Representative bright-field microscope images of T cells after 3 days of treatment with immobilized CD3/CD28 antibodies, αCD3scFv, and αCD28scFv polymers at a concentration of 3 nM. The corresponding fold expansion of T cells is shown in (**B**). (**C**) Representative images of T cells after 3 days of treatment with the indicated antibodies or polymer compositions (3 nM 5×αCD3scFv/1 nM 13×αCD28scFv, or 3 nM 13×αCD3scFv/1 nM 13×αCD28scFv). The corresponding fold expansion of T cells is shown in (**D**). (**E**) Fold expansion of T cells after 7 days of treatment with immobilized CD3/CD28 antibodies or SBCS using different concentrations of 13×αCD3scFv and 13×αCD28scFv. (**F**) Representative images of the T cells cultured with immobilized CD3/CD28 antibodies or SBCS (30 nM 13×αCD3scFv/10 nM 13×αCD28scFv) at day 7. (**G**) Representative images of the T cells cultured with immobilized CD3/CD28 antibodies or SBCS at day 14. (**H**) Fold expansion of T cells after 14 days of treatment with immobilized CD3/CD28 antibodies or SBCS. (**I**, **J**) CD4-to-CD8 ratio of CD4^+^ and CD8^+^ single-positive cells among live cells after treatment with CD3/CD28 immobilized antibodies or SBCS for 14 days. ‘d0’ represents peripheral blood mononuclear cells before cell expansion. Data in B, D, E, H and J represent mean ± s.d. of n = 3 healthy donors and are representative of at least three independent experiments. ** P < 0.01, *** P < 0.001. ns, not significant. Scale bars = 100 μm.

To evaluate the expansion of primary human T cells stimulated by the polymers, the T cells were cultured with 5×αCD3scFv, 13×αCD3scFv, or 13×αCD28scFv for 3 days. As a result, distinct T cell clusters were observed in the groups treated with 5×αCD3scFv or 13×αCD3scFv rather than 13×αCD28scFv (Figure 2A). Approximately 2-fold expansion of T cells was obtained in 5×αCD3scFv cultures, and more than 3-fold greater expansion was obtained in immobilized CD3/CD28 antibodies or 13×αCD3scFv cultures than that in the IL-2 control group (Figure 2B). T cells were also cultured with 5×αCD3scFv/13×αCD28scFv or 13×αCD3scFv/13×αCD28scFv for 3 days. Compared with T cells treated with 5×αCD3scFv/13×vCD28scFv, those treated with 13×αCD3scFv/13×αCD28scFv led to larger T cell clusters and nearly 1.5- fold greater expansion (Figure 2C and 2D). Thus, 13×αCD3scFv/13×αCD28scFv was used for the subsequent experiments and henceforth referred to as SBCS. Furthermore, T cells were stimulated by SBCS using different concentrations of 13×αCD3scFv and 13×αCD28scFv for 7 days. As a result, 75-fold expansion of T cells was achieved in SBCS cultures, while 55-fold expansion was obtained in immobilized CD3/CD28 antibody cultures. In addition, higher concentration of 13×αCD3scFv enhanced the expansion of T cells while 13×αCD28scFv failed to promote the proliferation of T cells (Figure 2E). On day 7, T cell clusters were more evident in the SBCS cultures than in the immobilized CD3/CD28 antibody cultures (Figure 2F). On day 14, expansion of T cells treated with SBCS and immobilized CD3/CD28 antibody were 950- and 640-fold, respectively (Figure 2G and 2H). There was no significant change in the CD4-to-CD8 ratio of expanded T cells (Figure. 2I and 2J).

### Proliferation of primary human T cells isolated from aged cancer patients

**Figure 3:**
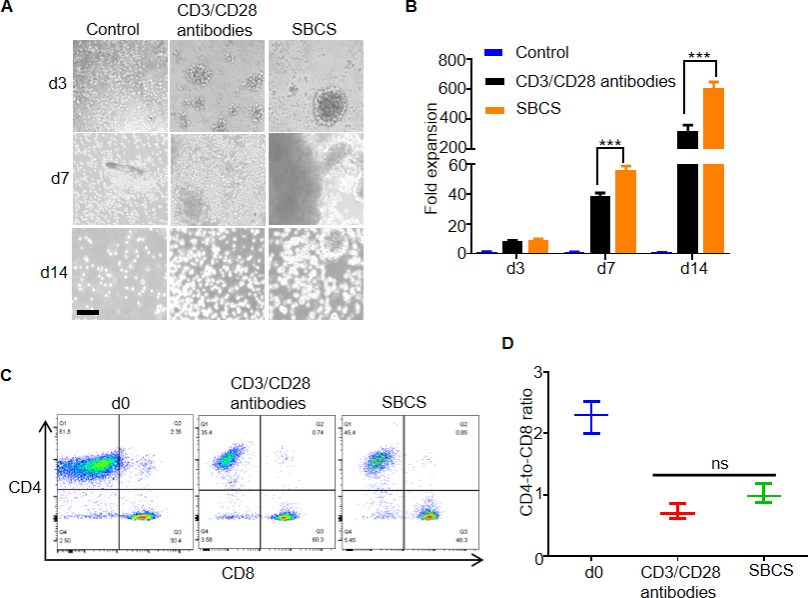
**Expansion of T cells from aged cancer patients.** (**A**) Representative images of T cells from aged cancer patients cultured with CD3/CD28 antibody or SBCS (30 nM 13×αCD3scFv/10 nM 13×αCD28scFv). (**B**) Fold expansion of T cells from aged cancer patients treated with immobilized CD3/CD28 antibodies or SBCS for 14 days. (**C**, **D**) CD4-to-CD8 ratio of CD4^+^ and CD8^+^ single-positive cells among live cells after treatment with CD3/CD28 immobilized antibodies or SBCS for 14 days. ‘d0’ represents peripheral blood mononuclear cells before cell expansion. Data in B–D represent mean ± s.d. of n = 3 aged cancer patients and are representative of at three independent experiments. *** P < 0.001. ns, not significant. Scale bars = 100 μm.

Next, we evaluated SBCS for the expansion of primary human T cells isolated from aged cancer patients. As shown in Figure 3A, the size and persistence of T cell clusters were greater in the SBCS cultures than in the immobilized CD3/CD28 antibody cultures. While more than 600-fold expansion of T cells was obtained in the SBCS cultures, only approximately 300-fold expansion was obtained in the CD3/CD28 antibody cultures on day 14 (Figure 3B). There was no significant change in the CD4-to-CD8 ratio among live T cells (Figure 3C and 3D).

### Antitumor efficacy of SBCS-prepared CAR-T cells

To evaluate whether T cells from aged cancer patients expanded with SBCS were functional *in vitro*, the expanded T cells were transfected with B7-H3-targeted CAR for preparing the B7-H3 CAR-T cells.

**Figure 4:**
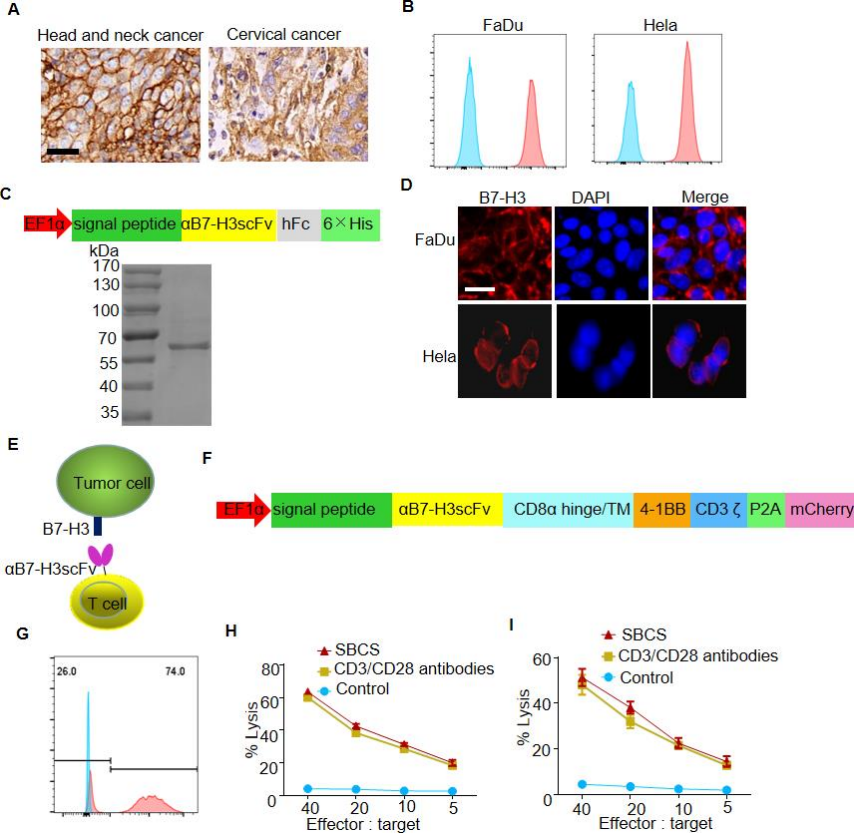
***In vitro* antitumor efficacy of B7-H3-targeted CAR-T cells produced with SBCS.** (**A**) Representative images of B7-H3 IHC staining in HNC and cervical cancer tissues. Scale bar, 20 μm. (**B**) Cell-surface expression of B7-H3 was evaluated by flow cytometry analysis. (**C**) Upper panel, schematic illustration of the vector for the expression of αB7-H3scFv-hFc. Lower panel, SDS-PAGE analysis of the purified αB7-H3scFv-hFc. (**D**) Immunofluorescent analysis of the expression of B7-H3 in FaDu and Hela cells using the recombinant αB7-H3scFv-hFc as the primary antibody. (**E**) Illustration of a B7-H3-targeted CAR-T cell against the tumor cell. (**F**) Schematic illustration of the vector for the expression of B7-H3 CAR. (**G**) The representative expression efficiency of B7-H3-redirected CAR on the T cells was evaluated using flow cytometry tracking the mCherry marker gene 10 days post-transfection. (**H**, **I**) Four-hour ^51^Cr release assays of B7-H3 CAR-T cells produced with SBCS or immobilized CD3/CD28 antibodies against FaDu or Hela cells. Scale bars = 50 μm. Data in G–H represent mean ± s.d. of three experimental replicates and are representative of at three experiments.

Recent studies reported that B7-H3 was overexpressed in multiple tumor tissues, especially in epithelial-derived tumor tissues including HNC and cervical cancer [16, 17]. In the present study, we firstly verified that B7-H3 was highly expressed in HNC and cervical cancer tissue by immunohistamycytosis, as well as on FaDu and Hela cell lines by flow cytometry (Figure 4A, 4B and Supplementary Table 1). Then, we constructed a vector for the expression of αB7-H3scFv-hFc, which was used to verify specific binding of αB7-H3scFv to B7-H3 (Figure 4C). As shown, FaDu and Hela cells were positively stained with B7-H3scFv-hFc as the primary antibody by immunofluorescence assay (Figure 4D).

Figure 4E shows that B7-H3 CAR-T cells targeted B7-H3 positive cells by specific binding of αB7-H3scFv to B7-H3. The lentivirus vector map of B7-H3-targeted CAR was shown in Figure 4F. The transfection efficiency of B7-H3 CAR against the SBCS expanded T cells was approximately 75% (Figure 4G). The transfection efficiency was similar in the CD3/CD28 antibody cultures (data not shown). The lysis rates of FaDu and Hela cells upon treatment with B7-H3 CAR-T cells were 20% and 15%, respectively, at the effector:target ratio of 5:1 (Figure 4H and 4I).

## DISCUSSION

In this study, we established a novel system (SBCS) for T cell expansion. In this system, the SunTag was used to recruit αCD3/CD28 scFv for forming αCD3scFv and αCD28scFv polymers, respectively, and the corresponding proteins could be easily obtained using eukaryotic expression systems.

As expected, T cell expansion was evident after treatment with αCD3scFv polymers, but not with αCD28scFv polymers. Our data also indicated that the proliferation of T cells mainly depended on the concentration of αCD3scFv polymers rather than that of αCD28scFv polymers. This observation can be explained based on the facts that the TCR/CD3 complex can be used as a key element to deliver signal 1 of T cell expansion and T cells can be expanded by CD3 antibodies alone [18]. Although CD3 antibody treatment alone provides a strong proliferative signal (signal 1) in the absence of costimulatory signals (signal 2), such proliferation is likely followed by premature T cell apoptosis or anergy [19]. CD3/CD28 antibodies to simultaneously deliver signals 1 and 2 can be used for T cell expansion without inducing early cell death [20]. In our study, the rate of T cell expansion with 5×αCD3scFv/13×αCD28scFv was lower than that with SBCS (13×αCD3scFv/13×αCD28scFv).Therefore, the repeat number of SunTag is also an important factor for the efficient activation of T cells. Notably, for the T cells from aged cancer patients, our SBCS system produced a 2-fold greater expansion than that obtained by conventional CD3/CD28 antibodies after 14-day stimulation. Moreover, it is difficult to expand the T cells from aged cancer patients for CAR-T therapy. Thus, compared with conventional methods, the SBCS T cell expansion system is more efficient, especially for T cells from aged cancer patients.

Unlike CD3/CD28 antibodies, SBCS is able to efficiently expand T cells without immobilization. SBCS consisting of recombined proteins can efficiently expand T cells under the stationary culture condition, while Dynabeads cannot. In addition, SBCS can be easily produced in most laboratories. Although the SBCS system has the advantages of low cost and high efficiency, there are some elements that should be optimized, such as the repeat number and linker length of the SunTag, as well as the ratio of αCD3scFv and CD28scFv polymers.

Rapid and scalable manufacture of functional T cells *in vitro* is a significant challenge in personalized T cell therapies. Here, we show that the low-cost SBCS system enables expansion of T cells with high efficiency, especially for T cells from aged cancer patients.

The expanded T cells can be used to prepare CAR-T cells, which showed potent antitumor efficacy *in vitro*. Thus, the SBCS method may be an attractive strategy for expansion of polyfunctional T cells.

## MATERIALS AND METHODS

### Cells and reagents

HEK293FT, FaDu, and Hela cell lines were purchased from the American Type Culture Collection. The cells were cultured with Dulbecco’s modified Eagle medium with 10% fetal bovine serum and 100 μg/mL penicillin–streptomycin. Polyethylenimine (PEI) used as a cell transfection reagent was purchased from Sigma (St. Louis, MO, USA). Antibodies used in this study included Cy3-conjugated goat anti-mouse IgG (ProteinTech, SA0009-1), PE mouse anti-human CD4 (BD Pharmingen, 55347), and FITC mouse anti-human CD8 (BD Pharmingen, 555366).

### Expression and purification of recombinant proteins

The sequences of anti-CD3 and anti-CD28 scFv (αCD3scFv and αCD28scFv) were obtained from the National Center for Biotechnology Information database and synthesized by GENEWIZ (Beijing, China). Fragments were subcloned into the vector pVax (Addgene, 74466) to construct plasmids pVax-αCD3scFv-4×GCN4, pVax-αCD3scFv-12×GCN4, pVax-αCD28scFv-12×GCN4, pVax-αGCN4scFv-αCD3scFv, and pVax-αGCN4scFv-αCD28scFv for recombinant protein expression. All the recombinant proteins were expressed in HEK293FT cells cultured with FreeStyle serum-free medium by transient transfection with PEI. Proteins were purified using Ni-NTA affinity columns and size exclusion chromatography. The concentration of stock protein solution was 1 mg/mL, and the proteins were store at -80°C until use.

### Expansion of T cells of healthy donors or aged cancer patients

Peripheral blood mononuclear cells were isolated from whole blood of healthy donors or aged cancer patients using Lymphoprep^TM^ (CORNING, 25-072-CL) gradient centrifugation (1000 g for 15 min at 25°C) according to the manufacturer’s instructions. Isolated PBMCs were initially cultured in X-VIVO^TM^ 15 serum-free hematopoietic cell medium (Lonza, 04-418Q) with recombinant human IL-2 (BBI Life Sciences) at a concentration of 100 units/mL. 13×αCD3scFv was assembled by mixing αCD3scFv-12×GCN4 and αGCN4scFv-αCD3scFv according to a mass proportion of 1:12 and incubating for 10 min at 37°C. 5×αCD3scFv and 13×αCD28scFv were prepared similarly. The traditional method using immobilized CD3 antibodies (5 μg/mL, Biolegend) and CD28 antibodies (5 μg/mL, Biolegend) followed a previously reported method [21]. T cell expansion by SBCS was performed with αCD3scFv polymers, αCD28scFv polymers, and IL-2 supplementation. Briefly, T cells (8 × 10^4^) were cultured in 96-well tissue culture plates containing X-VIVO Medium (Lonza) supplemented with activation stimulus (immobilized CD3/CD28 antibodies, αCD3scFv polymers or αCD28scFv polymers) and 100 U/mL IL-2 at 37°C in a humidified atmosphere with 5% CO2. Fresh medium with IL-2 was added every 3 days and maintain cells below a density of 2.5 × 10^6^ cells/ml throughout the culture period. T cell culture with only IL-2 supplementation was taken as the control group. Live cells were counted manually with a hemocytometer using the trypan blue exclusion method. T cell phenotype was evaluated using flow cytometry. Fold expansion was calculated by dividing the number of cells at the respective time point by the initial number of cells (8 × 10^4^).

### Antitumor efficacy of SBCS-prepared CAR-T cells

A total of 103 samples, including 62 HNC and 41 cervical cancer samples, were collected from West China Hospital. Immunohistochemistry (IHC) and flow cytometry assay were performed with an anti-B7-H3 rabbit mAb (Cell Signaling Technology; 1:200) and an anti-B7-H3 mouse mAb (BioLegend; 1:400), respectively. The anti-B7-H3 scFv (αB7-H3scFv) derived from 8H9 [22] was synthesized by GENEWIZ. αB7-H3scFv-hFc recombinant protein was expressed and purified in a eukaryotic expression system according to the method noted above. Immunofluorescence analysis with the αB7-H3scFv-hFc as the primary antibody was used to verify the binding of αB7-H3scFv to B7-H3 on FaDu or Hela cells.

A sequence encoding B7-H3-targeted CAR was synthesized by GENEWIZ. B7-H3-CAR and vehicle lentiviral vectors were produced using HEK293T cells according to previously published study [23]. In brief, cells were plated 24 h before transfection, then cotransfected with lentiviral constructs (B7-H3-CAR or vehicle-treated control vectors) and packaging plasmids (psPAX2 and pMD2.G vectors) using polyetherimide. Cell supernatants were harvested at 48 h and 72 h after transfection and then further concentrated 50-fold by centrifugation at 15,000 rpm for 2 h at 4°C. The pellet was resuspended in serum-free RPMI medium.

T cells isolated from aged cancer patients were expanded using SBCS or immobilized CD3/CD28 antibodies in X-VIVO Medium as the above method. Activated T cells were transduced with lentivirus (multiplicity of infection, MOI = 3–10) to express anti-B7-H3 CAR. Ten days after transfection, the expression efficiency of B7-H3-redirected CAR was evaluated by flow cytometry. Vehicle-transfected T cells were set as control.

Cytotoxic activity of B7-H3 CAR T cells was assessed using a standard ^51^Cr release assay [24]. The percentage of specific lysis was calculated using the following formula: (test release – spontaneous release) / (maximal release – spontaneous release) × 100.

### Statistical analysis

Data were expressed as mean ± standard deviation (SD) from three independent experiments. Statistical analyses were performed using GraphPad Prism Software version 5.0 (GraphPad Software, San Diego, CA, USA). Results were analyzed using Student’s *t* test. P < 0.05 was considered statistically significant.

### Ethic approval

The project protocol was approved by the Biomedical Ethics Committee of West China Hospital of Sichuan University. The use of tumor samples and blood samples from patients or donors was approved by the West China Hospital of Sichuan University Biomedical Ethics Committee (ethical approval document 2018-061). Written informed consent was obtained from patients and donors.

## Supplementary Material

Supplementary Table 1

## References

[1] June CH, Riddell SR, Schumacher TN. Adoptive cellular therapy: a race to the finish line. Sci Transl Med. 2015; 7:280ps7. 10.1126/scitranslmed.aaa3643 25810311

[2] Kantarjian H, Stein A, Gökbuget N, Fielding AK, Schuh AC, Ribera JM, Wei A, Dombret H, Foà R, Bassan R, Arslan Ö, Sanz MA, Bergeron J, et al. Blinatumomab versus chemotherapy for advanced acute lymphoblastic leukemia. N Engl J Med. 2017; 376:836–47. 10.1056/NEJMoa1609783 28249141 PMC5881572

[3] Maude SL, Frey N, Shaw PA, Aplenc R, Barrett DM, Bunin NJ, Chew A, Gonzalez VE, Zheng Z, Lacey SF, Mahnke YD, Melenhorst JJ, Rheingold SR, et al. Chimeric antigen receptor T cells for sustained remissions in leukemia. N Engl J Med. 2014; 371:1507–17. 10.1056/NEJMoa1407222 25317870 PMC4267531

[4] Huppa JB, Davis MM. T-cell-antigen recognition and the immunological synapse. Nat Rev Immunol. 2003; 3:973–83. 10.1038/nri1245 14647479

[5] Andersen PS, Menné C, Mariuzza RA, Geisler C, Karjalainen K. A response calculus for immobilized T cell receptor ligands. J Biol Chem. 2001; 276:49125–32. 10.1074/jbc.M109396200 11592972

[6] González PA, Carreño LJ, Coombs D, Mora JE, Palmieri E, Goldstein B, Nathenson SG, Kalergis AM. T cell receptor binding kinetics required for T cell activation depend on the density of cognate ligand on the antigen-presenting cell. Proc Natl Acad Sci USA. 2005; 102:4824–29. 10.1073/pnas.0500922102 15772168 PMC555720

[7] Yu B, Kusmartsev S, Cheng F, Paolini M, Nefedova Y, Sotomayor E, Gabrilovich D. Effective combination of chemotherapy and dendritic cell administration for the treatment of advanced-stage experimental breast cancer. Clin Cancer Res. 2003; 9:285–94. 12538481

[8] Trickett A, Kwan YL. T cell stimulation and expansion using anti-CD3/CD28 beads. J Immunol Methods. 2003; 275:251–55. 10.1016/s0022-1759(03)00010-3 12667688

[9] Uberti JP, Joshi I, Ueda M, Martilotti F, Sensenbrenner LL, Lum LG. Preclinical studies using immobilized OKT3 to activate human T cells for adoptive immunotherapy: optimal conditions for the proliferation and induction of non-MHC-restricted cytotoxicity. Clin Immunol Immunopathol. 1994; 70:234–40. 10.1006/clin.1994.1034 8313660

[10] Cheung AS, Zhang DK, Koshy ST, Mooney DJ. Scaffolds that mimic antigen-presenting cells enable ex vivo expansion of primary T cells. Nat Biotechnol. 2018; 36:160–69. 10.1038/nbt.4047 29334370 PMC5801009

[11] Tanenbaum ME, Gilbert LA, Qi LS, Weissman JS, Vale RD. A protein-tagging system for signal amplification in gene expression and fluorescence imaging. Cell. 2014; 159:635–46. 10.1016/j.cell.2014.09.039 25307933 PMC4252608

[12] Zhang X, Wang W, Shan L, Han L, Ma S, Zhang Y, Hao B, Lin Y, Rong Z. Correction to: gene activation in human cells using CRISPR/Cpf1-p300 and CRISPR/Cpf1-SunTag systems. Protein Cell. 2019; 10:776–77. 10.1007/s13238-018-0585-9 30417224 PMC6776487

[13] Pflueger C, Tan D, Swain T, Nguyen T, Pflueger J, Nefzger C, Polo JM, Ford E, Lister R. A modular dCas9-SunTag DNMT3A epigenome editing system overcomes pervasive off-target activity of direct fusion dCas9-DNMT3A constructs. Genome Res. 2018; 28:1193–206. 10.1101/gr.233049.117 29907613 PMC6071642

[14] Neguembor MV, Sebastian-Perez R, Aulicino F, Gomez-Garcia PA, Cosma MP, Lakadamyali M. (Po)STAC (polycistronic SunTAg modified CRISPR) enables live-cell and fixed-cell super-resolution imaging of multiple genes. Nucleic Acids Res. 2018; 46:e30. 10.1093/nar/gkx1271 29294098 PMC5861460

[15] Ye H, Rong Z, Lin Y. Live cell imaging of genomic loci using dCas9-SunTag system and a bright fluorescent protein. Protein Cell. 2017; 8:853–55. 10.1007/s13238-017-0460-0 28828720 PMC5676592

[16] Li Y, Zhang J, Han S, Qian Q, Chen Q, Liu L, Zhang Y. B7-H3 promotes the proliferation, migration and invasiveness of cervical cancer cells and is an indicator of poor prognosis. Oncol Rep. 2017; 38:1043–50. 10.3892/or.2017.5730 28627681

[17] Hu J, Jiang C, Zheng M, Guo Y, Tang X, Ren J, Lu D, Yu L, Gan W, Liu S, Tong A, Yang H. Overexpression of B7-H3 as an opportunity for targeted therapy in head and neck cancers. Am J Transl Res. 2019; 11:5183–96. 31497233 PMC6731436

[18] Parren PW, Geerts ME, Boeije LC, Aarden LA. Induction of t-cell proliferation by recombinant mouse and chimeric mouse/human anti-CD3 monoclonal antibodies. Res Immunol. 1991; 142:749–63. 10.1016/0923-2494(91)90121-x 1839077

[19] Schwartz RH. A cell culture model for T lymphocyte clonal anergy. Science. 1990; 248:1349–56. 10.1126/science.2113314 2113314

[20] Vonderheide RH, June CH. A translational bridge to cancer immunotherapy: exploiting costimulation and target antigens for active and passive T cell immunotherapy. Immunol Res. 2003; 27:341–56. 10.1385/IR:27:2-3:341 12857980

[21] Yamada-Ohnishi Y, Azuma H, Urushibara N, Yamaguchi M, Fujihara M, Kobata T, Ikeda H. Cytotoxic difference of T cells expanded with anti-CD3 monoclonal antibody in the presence and absence of anti-CD 28 monoclonal antibody. Stem Cells Dev. 2004; 13:315–22. 10.1089/154732804323099244 15186727

[22] Ahmed M, Cheng M, Zhao Q, Goldgur Y, Cheal SM, Guo HF, Larson SM, Cheung NK. Humanized affinity-matured monoclonal antibody 8H9 has potent antitumor activity and binds to FG loop of tumor antigen B7-H3. J Biol Chem. 2015; 290:30018–29. 10.1074/jbc.M115.679852 26487718 PMC4705981

[23] Tang X, Zhao S, Zhang Y, Wang Y, Zhang Z, Yang M, Zhu Y, Zhang G, Guo G, Tong A, Zhou L. B7-H3 as a novel CAR-T therapeutic target for glioblastoma. Mol Ther Oncolytics. 2019; 14:279–87. 10.1016/j.omto.2019.07.002 31485480 PMC6713854

[24] Wierda WG, Mehr DS, Kim YB. Comparison of fluorochrome-labeled and 51Cr-labeled targets for natural killer cytotoxicity assay. J Immunol Methods. 1989; 122:15–24. 10.1016/0022-1759(89)90329-3 2760476

