# Supplementary material for: T cell stimulation and expansion by SunTag-based clustering of anti-CD3/CD28 scFv
Source: Aging (Albany NY). 2020 Jun 10;12(11):11061–70. doi: 10.18632/aging.103318 (PMC7346064; doi:10.18632/aging.103318)
Supplement: Supplementary Table 1 [file aging-12-103318-s001..pdf]

SUPPLEMENTARY TABLE

Supplementary Table 1. B7-H3 expression patterns in HNC and cervical cancer tissues analyzed with IHC.

Tumor Type	Stained	Positive	Intensity			
			–	+	++	+++
HNC	62	65%	22	10	12	18
Cervical cancer	41	73%	12	5	8	16

Note: The staining intensity was scored using a common four-point scale as follows: no expression (–), <20% positive cells (+, low or weak expression), 20–50% positive cells (++, moderate expression), and >50% positive cells (+++, high or strong expression).
HNC: Head and neck cancer
